# Supplementary material for: End-to-end multimodal 3D imaging and machine learning workflow for non-destructive phenotyping of grapevine trunk internal structure
Source: Sci Rep. 2024 Feb 29;14:5033. doi: 10.1038/s41598-024-55186-3 (PMC10904756; doi:10.1038/s41598-024-55186-3)
Supplement: Supplementary file 1 — Supplementary Information. [file 41598_2024_55186_MOESM1_ESM.pdf]

SUPPLEMENTARY FIGURES AND TABLES

End-to-end multimodal 3D imaging and machine learning workflow for non-destructive phenotyping of grapevine trunk internal structure

AUTHORS

Romain Fernandez[1,2,10]*, Loïc Le Cunff[1,10], Samuel Mérigeaud[3], Jean-Luc Verdeil[4,10], Julie Perry[5], Philippe Larignon[6], Anne-Sophie Spilmont[7], Philippe Chatelet[10], Maïda Cardoso[8], Christophe Goze- Bac[9], and Cédric Moisy[1,10]*

AFFILIATIONS

[1] IFV, French Institute of Vine and Wine, UMT Géno-Vigne, IFV, INRAE, Institut Agro, F-34398 Montpellier, France.
[2] CIRAD, UMR AGAP Institut, F-34398 Montpellier, France.
[3] Tridilogy, Groupe CRP - Imaneo, Montpellier, France.
[4] CIRAD, Phiv, Campus Lavalette, 389 Avenue Agropolis, Montferrier-sur-Lez, France.
[5] CIVC Comité Champagne, 5 rue Henri Martin, 51200 Epernay, France.
[6] IFV Nîmes. Pôle Rhône-Méditerranée, 7 avenue Cazeaux, 30230 Rodilhan, France
[7] IFV Pôle Matériel Végétal, Domaine de l'Espiguette, 30240 Le Grau du Roi, France.
[8] BNIF University of Montpellier, Place Eugène Bataillon, Montpellier, France.
[9] Laboratoire Charles Coulomb, University of Montpellier and CNRS, 34095 Montpellier, France
[10] UMR AGAP Institut, Univ Montpellier, CIRAD, INRAE, Institut Agro, Montpellier, France.
* These authors contributed equally to this work.

Corresponding author: Cédric Moisy
Email: cedric.moisy@vignevin.com Tel: +33(0)467614471

SUPPORTING INFORMATION

The following supporting information is available for this article:

Vine #	Background	Classes<br>Intact tissues	Degraded tissues	White rot	Bark	Total samples	%
**01**	683	3,749	2,699	155	560	**7,846**	*9.6*
**02**	781	3,079	2,490	337	599	**7,286**	*8.9*
**03**	252	2,231	2,106	49	528	**5,166**	*6.3*
**04**	538	2,290	3,155	1,450	550	**7,983**	*9.8*
**05**	135	2,013	2,690	2,681	192	**7,711**	*9.5*
**06**	272	2,168	2,821	1,250	301	**6,812**	*8.4*
**07**	317	2,743	2,343	1,169	451	**7,023**	*8.6*
**08**	461	2,179	2,247	665	319	**5,871**	*7.2*
**09**	288	2,868	1,500	479	559	**5,694**	*7.0*
**10**	403	2,154	2,578	2,167	180	**7,482**	*9.2*
**11**	192	2,004	2,755	894	358	**6,203**	*7.6*
**12**	274	1,123	2,733	2,009	238	**6,377**	*7.8*
**Total**	**4,596**	**28,601**	**3,0117**	**13,305**	**4,835**	**81,454**	*100*
%	*5.6*	*35.1*	*37.0*	*16.3*	*5.9*	*100*	

Table S1 Number of annotated samples available for classifier training and evaluation
Distribution among tissue classes and vines.

Table S2 Evaluation of classifier performances
Global and class accuracies (Acc), precision (Prec), recall (Rec) and F1-scores (F1) percentages. Mean (bold) and standard deviation (*italic*) were collected by training on ten vines and evaluating on the last two. Different combinations of imaging modalities were tested: MRI PD-w, T1-w, and T2-w; and X-ray CT (XR).

Mean *Std*	Imaging modalities considered				Global	Background				Intact				Degraded				White rot				Bark			
					Acc	Acc	Prec	Rec	F1	Acc	Prec	Rec	F1	Acc	Prec	Rec	F1	Acc	Prec	Rec	F1	Acc	Prec	Rec	F1
MRI only	PD				**64.1**	**92.8**	**44.8**	**52.2**	**48.2**	**86.3**	**82.1**	**76.9**	**79.4**	**69.8**	**59.5**	**67.6**	**63.3**	**85.4**	**55.0**	**46.2**	**50.2**	**93.9**	**50.3**	**38.4**	**43.5**
					8.7	*2.9*	*19.2*	*15.4*	*17.1*	*5.8*	*8.6*	*18.0*	*11.6*	*7.4*	*9.7*	*5.8*	*7.2*	*5.3*	*18.8*	*18.6*	*18.7*	*1.6*	*18.3*	*12.7*	*15.0*
		T1			**76.4**	**95.0**	**68.3**	**79.9**	**73.7**	**91.4**	**84.9**	**90.8**	**87.8**	**80.5**	**74.7**	**72.7**	**73.7**	**89.7**	**70.9**	**61.9**	**66.1**	**96.3**	**71.0**	**63.9**	**67.2**
					9.8	*5.5*	*25.9*	*8.0*	*12.2*	*3.1*	*8.4*	*8.8*	*8.6*	*6.6*	*9.1*	*7.9*	*8.4*	*6.0*	*13.5*	*21.0*	*16.4*	*1.5*	*16.7*	*12.7*	*14.4*
			T2		**73.2**	**93.5**	**41.7**	**37.9**	**39.7**	**94.1**	**89.7**	**94.0**	**91.8**	**75.3**	**66.0**	**73.2**	**69.4**	**87.8**	**63.1**	**46.4**	**53.5**	**95.8**	**66.6**	**56.6**	**61.2**
					5.5	*1.5*	*14.8*	*15.7*	*15.2*	*2.1*	*4.4*	*6.1*	*5.1*	*5.7*	*7.8*	*5.6*	*6.5*	*5.7*	*14.9*	*20.4*	*17.2*	*1.1*	*15.1*	*13.3*	*14.1*
		T1	T2		**80.2**	**94.8**	**66.6**	**78.5**	**72.1**	**95.5**	**91.9**	**95.8**	**93.8**	**83.8**	**77.8**	**80.3**	**79.0**	**89.6**	**72.7**	**57.3**	**64.1**	**96.7**	**77.0**	**64.8**	**70.4**
					7.6	*5.7*	*25.2*	*8.9*	*13.1*	*1.6*	*2.8*	*4.1*	*3.3*	*4.5*	*7.7*	*5.2*	*6.2*	*6.2*	*12.3*	*21.6*	*15.6*	*1.5*	*14.7*	*12.0*	*13.2*
	PD	T1			**75.1**	**94.9**	**68.4**	**77.3**	**72.5**	**91.6**	**85.4**	**91.1**	**88.2**	**78.9**	**70.7**	**74.0**	**72.3**	**88.8**	**70.8**	**54.6**	**61.6**	**96.0**	**70.5**	**55.4**	**62.0**
					8.1	*5.4*	*26.0*	*8.5*	*12.8*	*2.2*	*7.4*	*6.2*	*6.7*	*5.2*	*7.8*	*5.8*	*6.7*	*6.0*	*13.8*	*20.5*	*16.5*	*1.2*	*18.4*	*16.3*	*17.3*
	PD		T2		**73.7**	**93.5**	**52.4**	**50.1**	**51.2**	**93.7**	**89.3**	**93.8**	**91.5**	**76.3**	**66.9**	**74.0**	**70.3**	**87.8**	**65.8**	**46.1**	**54.2**	**96.0**	**70.6**	**58.0**	**63.7**
					5.7	*2.7*	*22.6*	*17.2*	*19.6*	*2.3*	*5.0*	*6.3*	*5.6*	*5.0*	*8.6*	*7.2*	*7.8*	*5.5*	*16.4*	*18.4*	*17.4*	*1.0*	*14.9*	*14.6*	*14.7*
	PD	T1	T2		**78.8**	**94.6**	**66.3**	**78.1**	**71.7**	**95.2**	**91.0**	**96.1**	**93.5**	**82.4**	**75.1**	**79.5**	**77.2**	**88.8**	**72.7**	**50.2**	**59.4**	**96.7**	**76.3**	**64.2**	**69.8**
					7.4	*5.9*	*25.3*	*8.8*	*13.1*	*1.9*	*3.8*	*4.0*	*3.9*	*4.5*	*7.9*	*5.9*	*6.7*	*6.1*	*11.8*	*20.4*	*14.9*	*1.1*	*14.5*	*12.7*	*13.5*
XR only				XR	**85.6**	**99.7**	**97.0**	**97.4**	**97.2**	**88.8**	**83.2**	**86.2**	**84.7**	**87.0**	**83.8**	**80.9**	**82.3**	**98.1**	**91.8**	**93.4**	**92.6**	**97.7**	**79.6**	**83.9**	**81.7**
					2.7	*0.2*	*4.0*	*2.3*	*2.9*	*2.6*	*6.7*	*5.2*	*5.9*	*2.6*	*5.9*	*6.3*	*6.1*	*0.5*	*9.2*	*2.9*	*4.4*	*1.2*	*9.1*	*6.6*	*7.7*
MRI and XR	PD			XR	**86.9**	**99.7**	**97.5**	**97.3**	**97.4**	**89.9**	**85.4**	**86.2**	**85.8**	**88.0**	**84.3**	**83.6**	**84.0**	**98.1**	**91.8**	**93.6**	**92.7**	**98.0**	**82.7**	**84.9**	**83.8**
					2.5	*0.2*	*2.6*	*2.3*	*2.5*	*2.6*	*7.6*	*6.2*	*6.8*	*2.4*	*5.6*	*6.6*	*6.0*	*0.5*	*9.1*	*2.8*	*4.3*	*0.7*	*7.2*	*6.4*	*6.8*
		T1		XR	**88.8**	**99.6**	**96.1**	**97.3**	**96.7**	**91.9**	**86.4**	**90.8**	**88.5**	**89.7**	**87.9**	**84.3**	**86.1**	**98.2**	**92.5**	**93.4**	**93.0**	**98.3**	**86.1**	**86.1**	**86.1**
					2.8	*0.3*	*5.4*	*2.2*	*3.1*	*2.4*	*7.0*	*8.5*	*7.7*	*2.3*	*5.2*	*5.5*	*5.4*	*0.7*	*9.3*	*3.5*	*5.1*	*0.8*	*8.5*	*6.8*	*7.6*
			T2	XR	**92.0**	**99.7**	**97.9**	**97.2**	**97.5**	**95.1**	**91.7**	**94.7**	**93.2**	**92.5**	**90.8**	**88.7**	**89.7**	**98.2**	**92.2**	**93.4**	**92.8**	**98.5**	**87.5**	**86.1**	**86.8**
					1.5	*0.2*	*2.4*	*2.2*	*2.3*	*1.7*	*3.5*	*5.1*	*4.1*	*1.5*	*3.6*	*3.5*	*3.5*	*0.4*	*9.2*	*3.0*	*4.5*	*0.6*	*6.5*	*5.6*	*6.0*
	PD	T1		XR	**88.5**	**99.6**	**96.2**	**97.3**	**96.7**	**91.6**	**86.2**	**90.4**	**88.2**	**89.4**	**87.2**	**84.2**	**85.7**	**98.1**	**92.5**	**92.9**	**92.7**	**98.3**	**86.6**	**85.2**	**85.9**
					2.1	*0.5*	*6.6*	*2.1*	*3.2*	*1.9*	*7.2*	*5.9*	*6.5*	*1.8*	*4.6*	*6.1*	*5.3*	*0.9*	*9.3*	*4.2*	*5.8*	*0.7*	*8.5*	*6.0*	*7.0*
	PD		T2	XR	**91.3**	**99.7**	**97.9**	**97.0**	**97.4**	**94.5**	**90.8**	**94.2**	**92.5**	**91.9**	**90.2**	**87.6**	**88.9**	**98.1**	**92.1**	**93.0**	**92.6**	**98.4**	**87.7**	**85.4**	**86.6**
					1.6	*0.2*	*2.1*	*2.2*	*2.1*	*1.9*	*4.3*	*5.8*	*4.9*	*1.6*	*4.3*	*5.0*	*4.6*	*0.5*	*9.2*	*3.1*	*4.6*	*0.5*	*7.0*	*5.9*	*6.4*
		T1	T2	XR	**92.1**	**99.3**	**91.5**	**96.9**	**94.2**	**95.6**	**92.3**	**95.6**	**93.9**	**93.0**	**91.5**	**89.5**	**90.5**	**97.7**	**92.3**	**91.4**	**91.9**	**98.4**	**87.3**	**86.0**	**86.7**
					1.8	*0.9*	*15.3*	*2.1*	*3.7*	*1.6*	*2.8*	*4.2*	*3.4*	*1.5*	*3.6*	*2.8*	*3.2*	*1.2*	*9.2*	*4.4*	*5.9*	*0.6*	*7.5*	*6.0*	*6.6*
	PD	T1	T2	XR	**91.6**	**99.2**	**90.5**	**96.8**	**93.5**	**95.3**	**91.4**	**95.9**	**93.6**	**92.7**	**91.5**	**88.6**	**90.0**	**97.5**	**92.3**	**90.5**	**91.4**	**98.4**	**87.3**	**85.5**	**86.4**
					2.0	*1.1*	*17.4*	*2.1*	*3.7*	*1.7*	*3.4*	*4.1*	*3.7*	*1.5*	*3.8*	*3.7*	*3.8*	*1.5*	*9.2*	*5.3*	*6.8*	*0.7*	*8.7*	*5.8*	*6.9*

	Sample #	Back-ground	Intact	Degraded	White rot	Bark	%
PREDICTED	Background	**48,824**	4	32	3,818	894	*6.0*
	Intact	0	**300,293**	24,096	0	1,065	*36.3*
	Degraded	31	13,611	**296,931**	9,085	5,276	*36.3*
	White rot	652	3	6,26	**132,996**	455	*15.7*
	Bark	1,049	700	3,968	456	**45,495**	*5.8*
	%	*5.6*	*35.1*	*37.0*	*16.3*	*5.9*	

(Column header group: REAL classes)

Table S3 Evaluation of the classifier performance: sum of confusion matrices
Considering the 66 folds of the cross-validation.

Imaging modalities	Tissue classes: Intact	Degraded	White rot
**X-ray**	**129.4**	**104.5**	**46.0**
	21.3	*24.6*	*14.1*
**T1-w**	**110.2**	**47.1**	**29.7**
	40.0	*42.6*	*19.7*
**T2-w**	**68.9**	**9.4**	**2.2**
	46.1	*20.4*	*3.0*
**PD-w**	**41.8**	**12.0**	**4.3**
	37.2	*17.4*	*6.8*

Table S4 Multimodal signal values corresponding to the three main tissue classes
Means (in bold) and standard deviations (italic) (values in 8-bits) collected on the whole dataset (after automatic classification, 46.2 million voxels total).

		Tissue content cm^3 (%)		
Vine #	Phenotype	Intact	Degraded	White rot
01	AS-A ASymptomatic Always	687 (2.0)	302 (29.9)	21 (68.0)
02	AS-A ASymptomatic Always	571 (4.2)	278 (31.3)	38 (64.3)
03	AS-A ASymptomatic Always	663 (1.7)	336 (33.0)	18 (65.1)
04	SY-N Symptomatic Neo	722 (9.5)	395 (31.9)	118 (58.4)
05	SY-N Symptomatic Neo	426 (7.9)	450 (47.2)	76 (44.7)
06	SY-N Symptomatic Neo	559 (9.1)	334 (33.9)	90 (56.8)
07	AS-R ASymptomatic Resilient	278 (19.2)	623 (55.8)	215 (24.9)
08	AS-R ASymptomatic Resilient	357 (23.6)	551 (46.3)	281 (30.0)
09	AS-R ASymptomatic Resilient	421 (10.8)	368 (41.5)	96 (47.5)
10	SY-A SYmptomatic Apoplectic	333 (20.8)	436 (44.9)	202 (34.2)
11	SY-A SYmptomatic Apoplectic	425 (12.2)	685 (54.1)	155 (33.5)
12	SY-A SYmptomatic Apoplectic	252 (17.5)	565 (57.0)	174 (25.4)

Table S5 Tissue contents per vine

Contents measured for each individual vine from the automatically segmented 3D datasets. Data were collected in the region ranging from the upper last 20 cm of the trunks to the first 5 cm of the branches. Results are expressed as volume (cm^3) and percentages.

	Sum_of_FS_occurrences	Nb_of_Years_since_1st_FS	Nb_of_Years_without_FS	White_rot_%	Degraded_%	Intact_%	Total_WR+D_content_%	White_rot _*vs*_Intact_ratio	Intact_*vs*_Total_WR+D_ratio	FS_ in_2013	FS_ in_2014	FS_ in_2015	FS_ in_2016	FS_ in_2017	FS_ in_2018	FS_ in_2019
Sum_of_FS_occurrences	1	0.91	-0.68	0.84	0.79	-0.87	0.87	0.82	-0.86	0.47	0.47	0.61	0.18	0.67	0.55	0.42
Nb_of_Years_since_1st_FS		1	-0.6	0.91	0.74	-0.87	0.87	0.88	-0.85	0.67	0.45	0.35	0.25	0.49	0.62	0.16
Nb_of_Years_without_FS			1	-0.69	-0.62	0.69	-0.69	-0.56	0.81	-0.25	-0.3	-0.2	-0.27	-0.31	-0.31	-0.66
White_rot_%				1	0.71	-0.9	0.9	0.96	-0.89	0.68	0.31	0.27	0.19	0.62	0.49	0.19
Degraded_%					1	-0.95	0.95	0.8	-0.92	0.15	0.33	0.46	0.5	0.62	0.36	0.27
Intact_%						1	-1	-0.94	0.98	-0.41	-0.35	-0.41	-0.39	-0.68	-0.44	-0.25
Total_WR+D_content_%							1	0.94	-0.98	0.41	0.35	0.41	0.39	0.68	0.44	0.25
White_rot _*vs*_Intact_ratio								1	-0.88	0.57	0.23	0.37	0.23	0.72	0.47	0.07
Intact_*vs*_Total_WR+D_ratio									1	-0.4	-0.37	-0.33	-0.42	-0.59	-0.44	-0.34
FS_ in_2013										1	0.4	-0.13	-0.26	0.26	0.26	0
FS_ in_2014											1	-0.13	0.26	0.26	-0.26	0.45
FS_ in_2015												1	-0.17	0.52	0.52	0.3
FS_ in_2016													1	0.11	0.11	-0.19
FS_ in_2017														1	0.11	0.19
FS_ in_2018															1	-0.19
FS_ in_2019																1

1, 0.75, 0.5, 0.25, 0, -0.25, -0.5, -0.75, -1

Table S6 Correlogram

Chart of correlation statistics between "internal" (blue text) and "external" (black text) proxies for GTD status diagnosis. FS = foliar symptom; Nb = number; D = degraded tissues; WR = white rot.

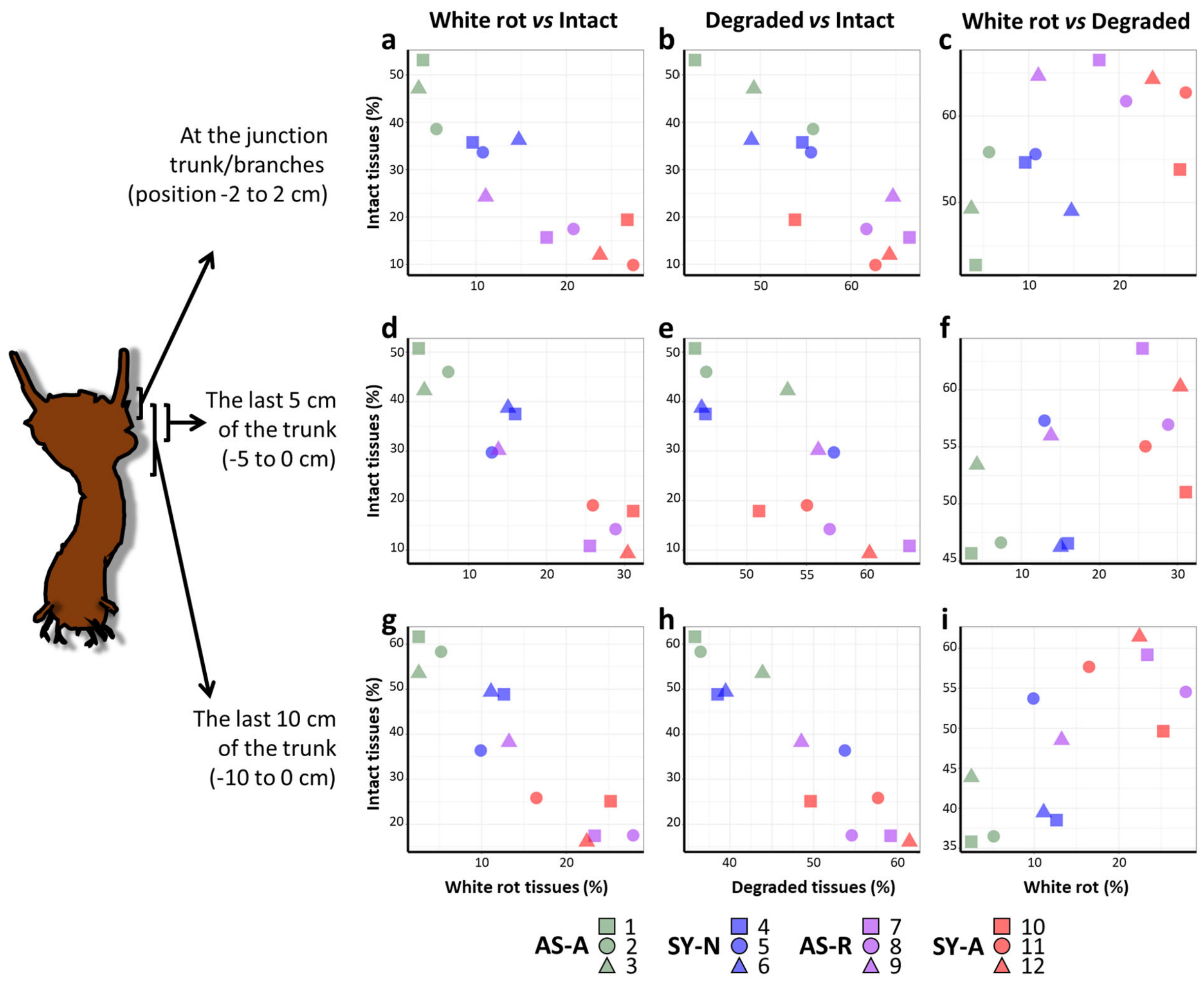

Fig. S1 Detailed comparison of vines for intact, degraded, and white rot contents considering different positions along the vine trunk

AS-A = asymptomatic-always; SY-N = symptomatic-neo; AS-R = asymptomatic-resilient; SY-A = symptomatic-apoplectic.

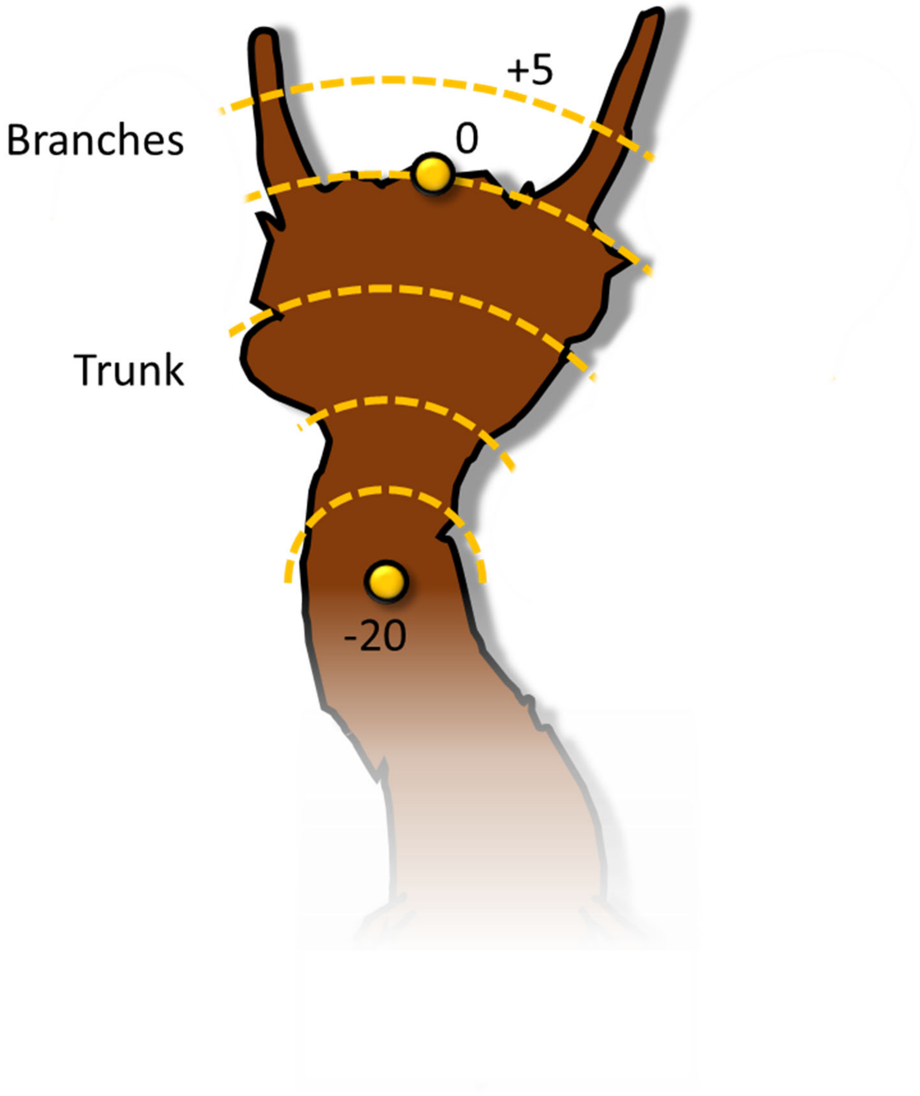

Fig. S2 Vine trunk geodesic distance estimation
Geodesic distances were estimated from the center of the trunk and using the top of the trunk as a reference (point "0").
